# Medical Advice on the Grand Scale

**Published:** 1907-06-22

**Authors:** 


### The Hospital

A Journal of

The Medical Sciences and Hospital Administration.

New Series. No. 13, Vol. I. [No. 1085, Vol. XLII.] SATURDAY, JUNE 22, 1907.

### Medical Advice on the Grand Scale.

The public nowadays has no need to complain of lack of advice on medical problems. But whether the numerous counsellors who so freely offer their gratuitous recommendations form a guarantee of safety is another question. It is possible that confusion, rather than knowledge, is the most prominent result of their efforts. We may put on one side the proprietors of nostrums, each of whom, very obviously, has an axe of his own to grind. Nor are we concerned with anonymous busybodies, who, as opportunity offers, proclaim their several views on disease and treatment. All these entail consequences which help to make sensible medical practice difficult, and are not without prejudice to the welfare of individual patients. They are, however, among the facts which exist, and must, for the time, be endured. But something more than protest is necessary when prominent and responsible members of the profession allow themselves to be led into public and sensational announcements that claim to be large generalisations and yet are quite inappropriate in individual instances. We are more than ready to recognise that medicine has a duty to educate public opinion on questions which concern the health of the community. But this is an entirely different matter from the increasing habit of discussing in the public prints technical professional problems which are full of difficulty, and which do not lend themselves to general propositions. The latest proclamation of this order is, that for people to take medicine, when they are sick, is an "extraordinary habit," which, in a more enlightened future, will certainly cease to be. The distinction of the orator who appears to be responsible for this statement will doubtless secure its extensive quotation, and there can be no doubt the proposition will be urged against many a proposed scheme of treatment. As a consequence, individual practitioners, with urgent responsibilities to patients under their care, will be restricted and handicapped in their therapeutic efforts. As for the statement itself, it is a manifest exaggeration. There are, of course, numerous daily instances in which the taking of appropriate medicines means the difference between pain and suffering, and sometimes even between life and death.

We will venture in this association to put in a claim for a recognition of the duties and responsibilities of the family medical practitioner, and to urge that these demand help, and not hindrance, from prominent and influential members of the profession. Whatever may be the case with others, it is certain that the general practitioner is helping to make medicine an effective and actual instrument of public service. Equipped, as he has to be, in many directions, he is carrying the ministry of his profession into the lives and homes of the great mass of the people. In doing this, he has difficulties of which those who are ignorant of the details of his work can have but a faint and inadequate perception. Apart from social, personal, and financial claims, his strictly technical and professional obligations have to be met in circumstances which make their faithful discharge a most anxious task. To him there come those early beginnings of disease, the true and confident interpretation of which is often beyond the resources of medical science; and yet on these public ignorance and impatience and natural anxiety combine to demand an immediate verdict. Through the numberless details of the treatment required in a long and troublesome illness, the family practitioner must prove himself an unwearied and resourceful guide, ready for all emergencies, patient of much stupidity and ignorance. In all this he is face to face with the individual patient, and it is personal peculiarities and special wants that he must estimate, and to these that he must adjust his remedial measures. Now this, we repeat, is a large and important part of medical service. It is at once the glory and the defence of professional existence. Is it reasonable and fair that such work should be hampered by *ex cathedrâ* utterances from those who know little of its nature and for whom the triumphs of the platform and the paragraph are appropriate rewards?

Further, as a matter of principle, we dissent from this practice of offering medical advice on the grand scale. The prevention of disease occupies quite a different position from the discussion of methods of treatment. Prevention can only be secured by the aid of public opinion acting through local or national representatives, and the principles and methods necessary for success are fairly defined and well established. It is highly appropriate, therefore, that these principles and methods shall be urged on the attention of the community. But treatment of actually existing disease is concerned with an individual sufferer. There is no room for public proclamations on the virtues of particular forms and methods of treatment. Let such questions, by all means, be discussed in our medical schools and societies, and let an accumulated experience have its due weight on the judgment of the practitioner. But in the last resort the decision must be founded on what seems best in the special circumstances of the individual patient, and an attempt to enforce a formula which shall restrict the freedom of personal judgment is opposed to free and responsible medical practice. Hence we have on former occasions protested against public lectures by practitioners on such subjects as the methods of infant feeding and the alcohol question. The latest effort is absurd enough in its quality, though no doubt it will be hailed as the word of a seer. If we must have "leaders," and they must talk nonsense, they might at least abstain from dangerous nonsense.

